# Bilateral vertebral artery dissection extending to the left posterior cerebral artery: A case report

**DOI:** 10.1016/j.radcr.2023.01.099

**Published:** 2023-02-27

**Authors:** Masahiro Morishita, Koichiro Shindo, Ryunosuke Yoshihara, Kohei Ishikawa, Ryota Nomura, Hideki Endo, Koji Oka, Hirohiko Nakamura

**Affiliations:** a Department of Neurosurgery, Nakamura Memorial South Hospital, 2-3-1 Kawazoe 2-jo, Minami-ku, Sapporo, Hokkaido 005-8555, Japan; b Department of Neurosurgery, Nakamura Memorial Hospital, South 1, West 14, Chuo-ku, Sapporo, Hokkaido 060-8570, Japan

**Keywords:** Intramural hematoma, Ischemic stroke, Magnetic resonance imaging, Vertebral artery dissection

## Abstract

Intracranial artery dissection accounts for a small percentage (1%-2%) of all ischemic strokes. Vertebral artery dissection sometimes extends to the basilar artery but very rarely to the posterior cerebral artery. We report a case of bilateral vertebral artery dissection extending to the left posterior cerebral artery with the characteristic distribution of intramural hematoma. A 51-year-old woman presented with right hemiparesis and dysarthria 3 days after sudden neck pain. Magnetic resonance imaging on admission revealed infarcts in the left thalamus and temporo-occipital lobe and findings suggestive of bilateral vertebral artery dissection. No infarct was detected in the brainstem. The patient was treated conservatively. Initially, we suspected that infarction in the left posterior cerebral artery territory had been caused by artery-to-artery embolism from the dissected vertebral arteries. However, T1-weighted imaging on day 15 of admission revealed intramural hematoma extending from the left vertebral artery to the left posterior cerebral artery. Therefore, we diagnosed bilateral vertebral artery dissection extending to the basilar artery and the left posterior cerebral artery. The patient's symptoms subsequently improved with conservative treatment, and she was discharged with a modified Rankin Scale score of 1 on day 62 of admission. In this case, intramural hematoma of the basilar artery was found in the anterior vessel wall. Brainstem infarction is less likely when intramural hematoma is located in the anterior vessel wall of the basilar artery in vertebrobasilar artery dissection. T1-weighted imaging is useful for the diagnosis of this rare condition and can predict potentially impaired branches and possible symptoms.

## Introduction

Intracranial artery dissection accounts for a small percentage (1%-2%) of all ischemic strokes [1], and intramural hematoma is a common finding. Previous studies reported that the morphology of intramural hematoma could be a predictor of cerebral infarction [2], [3], [4]. However, little is known about the relationship between the distribution of intramural hematoma and the symptoms and prognosis. Vertebral artery dissection sometimes extends to the basilar artery but very rarely to the posterior cerebral artery. Additionally, infarction in the territory of the posterior cerebral artery is thought to be caused more often by hemodynamic failure or artery-to-artery embolism from the dissected vertebral artery or basilar artery than by posterior cerebral artery dissection itself. We report a case of bilateral vertebral artery dissection extending to the left posterior cerebral artery and discuss the relationship between the distribution of intramural hematoma and symptoms and prognosis.

## Case report

**Fig. 1:**
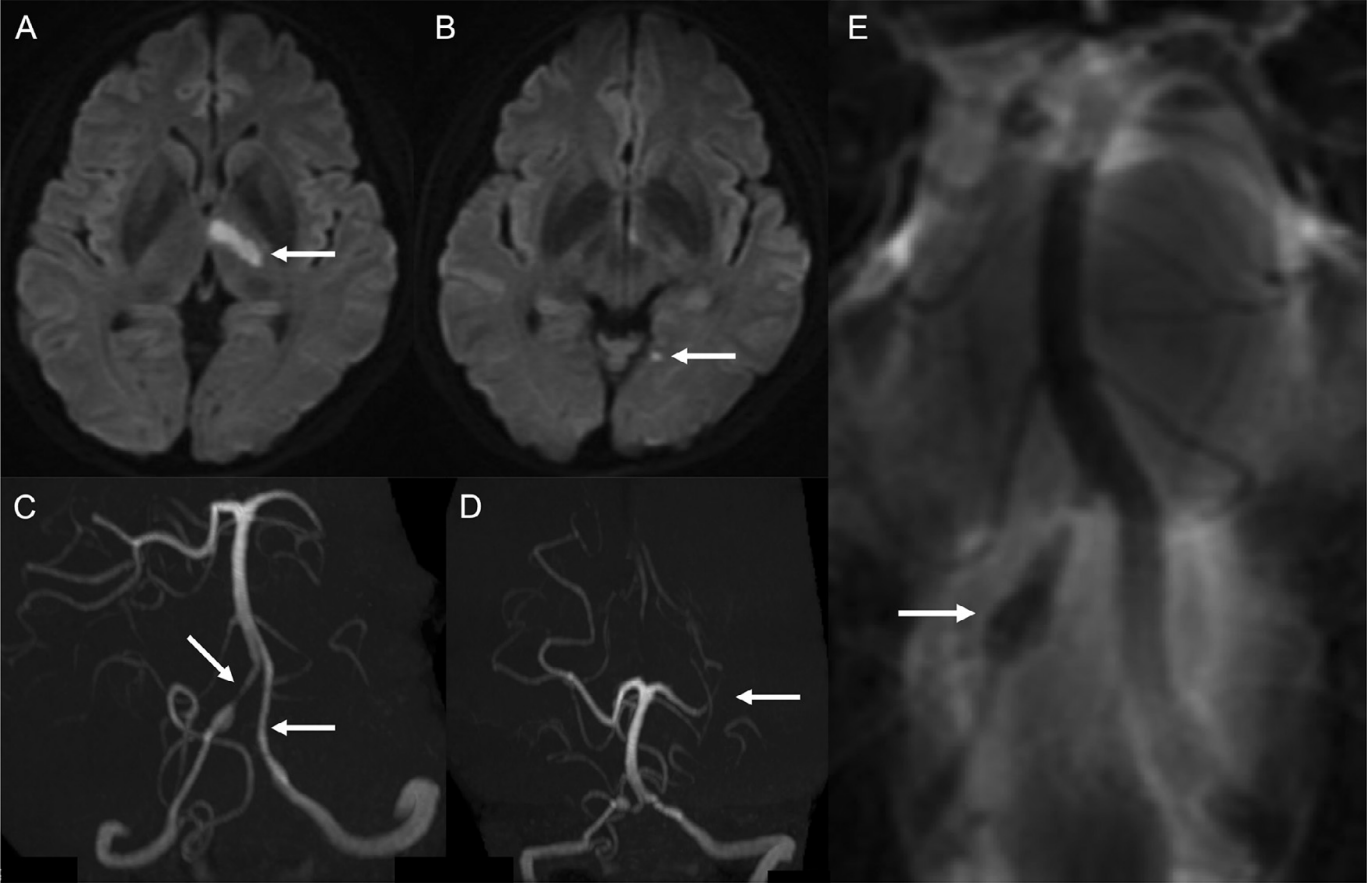
Magnetic resonance imaging on admission showing infarcts (arrows) in the left thalamus (A) and temporo-occipital lobe (B). Magnetic resonance angiography showing bilateral vertebral artery stenosis (arrows) (C) and posterior cerebral artery stenosis (arrow) (D). Basi-parallel anatomical scanning showing fusiform dilation of the right vertebral artery (arrow) (E).

**Fig. 2:**
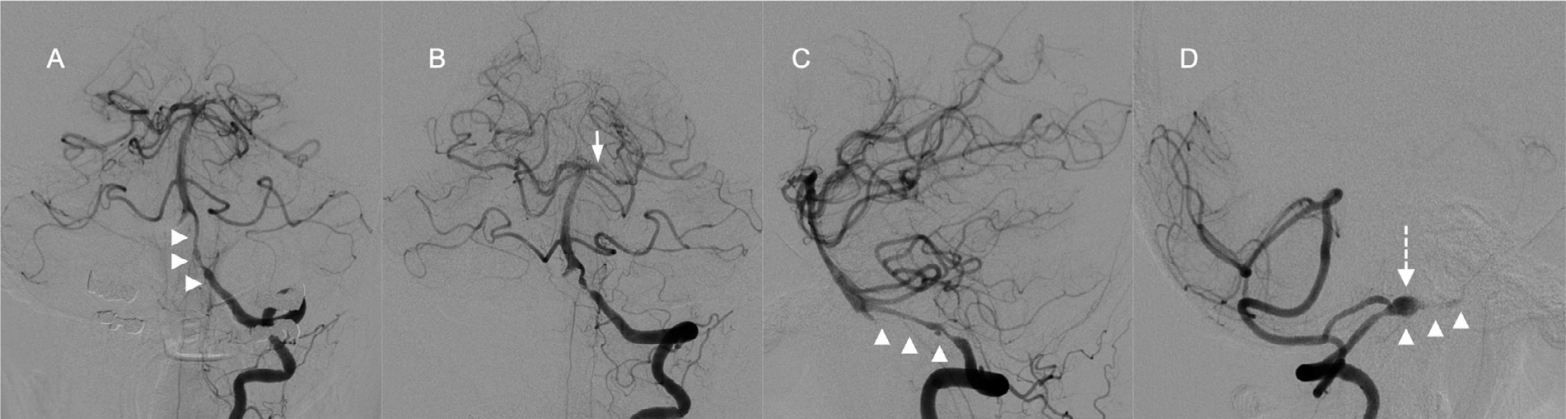
Digital subtraction angiography on day 6 of admission showing the pearl and string sign in bilateral vertebral arteries (arrow heads), stenosis of the P1 segment of the left posterior cerebral artery (arrow), and a right vertebral artery dissecting aneurysm involving the posterior inferior cerebellar artery origin (dotted arrow). Left vertebral angiograms: posteroanterior view (A), Townes view (B), and lateral view (C), and right vertebral angiogram, lateral view (D).

**Fig. 3:**
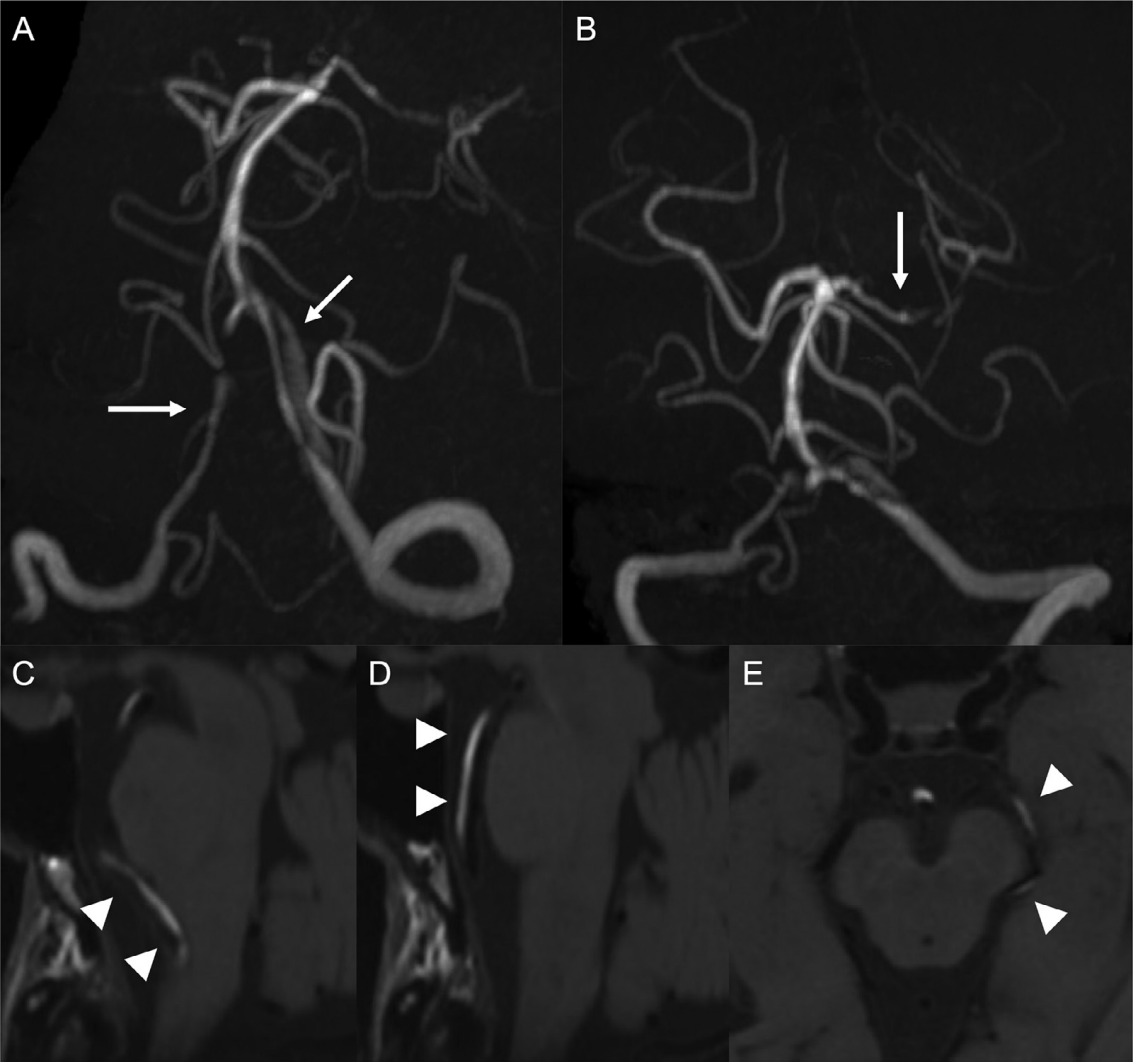
Magnetic resonance imaging on day 15 of admission showing progressive stenosis of bilateral vertebral arteries (arrows) (A) and the left posterior cerebral artery (arrow) (B). Intramural hematoma was visible as a hyperintense region (arrow heads) along the left vertebral artery (C), basilar artery (D), and left posterior cerebral artery (E) on T1-weighted imaging.

**Fig. 4:**
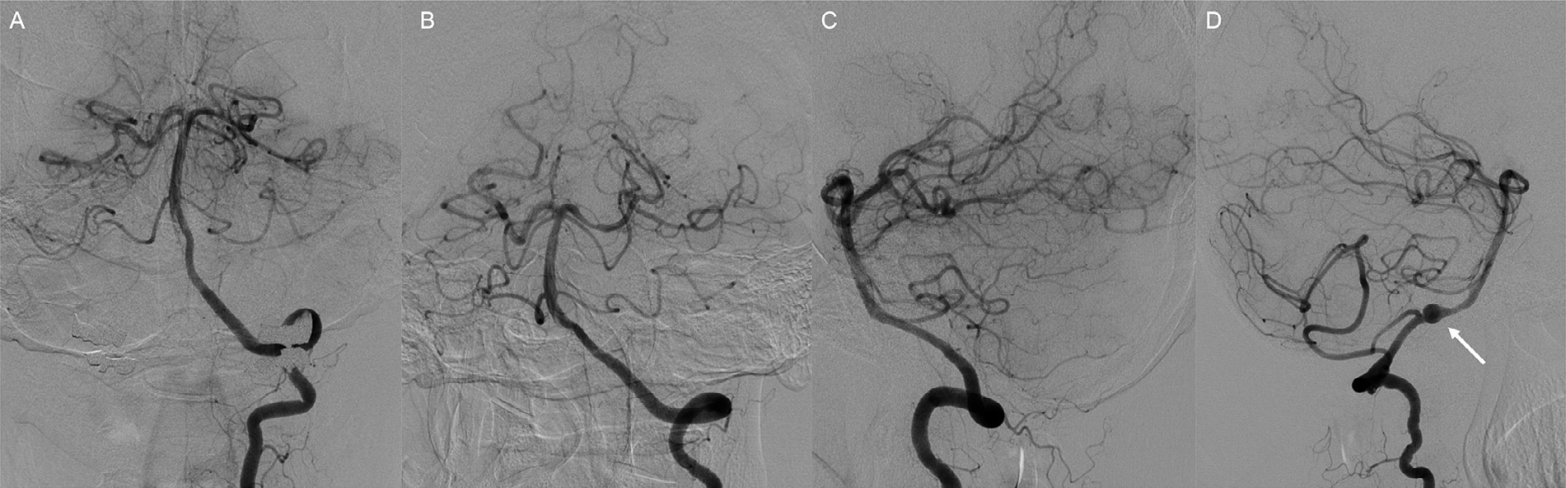
Digital subtraction angiography 6 months after onset showing that the diameter of the dissected arteries has returned to normal except for a dissecting aneurysm of the right vertebral artery (arrow). Left vertebral angiograms: posteroanterior view (A), Townes view (B), and lateral view (C), and right vertebral angiogram, lateral view (D).

A 51-year-old woman presented with right hemiparesis and dysarthria 3 days after sudden neck pain. Her initial National Institutes of Health Stroke Scale score was 5. Magnetic resonance imaging on admission showed infarcts in the territory of the left posterior cerebral artery (Figs. 1A and B). No infarct was detected in the brainstem. Magnetic resonance angiography showed bilateral vertebral artery stenosis and left posterior cerebral artery stenosis (Figs. 1C and D). Basi-parallel anatomic scanning revealed fusiform dilation of the right vertebral artery (Fig. 1E). Digital subtraction angiography (DSA) revealed bilateral vertebral artery dissection and no evidence of dissection in the extracranial vertebral arteries. DSA also showed stenosis of the P1 segment of the left posterior cerebral artery (Fig. 2). We suspected that infarction in the posterior cerebral artery territory had been caused by artery-to-artery embolism from the dissected vertebral arteries. Therefore, we initiated strict blood pressure control and antithrombotic treatment with argatroban infusion followed by cilostazol. Magnetic resonance imaging on day 15 of admission detected intramural hematoma along the left vertebral artery to the left posterior cerebral artery on T1-weighted imaging (T1WI) (Fig. 3). In particular, intramural hematoma was located in the anterior vessel wall of the basilar artery (Fig. 3D). The patient was diagnosed with bilateral vertebral artery dissection extending to the basilar artery and the left posterior cerebral artery on the basis of these imaging findings. Mild disturbance of consciousness and diplopia were noted after admission; however, these symptoms subsequently improved. The patient was discharged with a modified Rankin Scale score of 1 on day 62 of admission, with no recurrence of stroke during the 6-month follow-up. DSA 6 months after discharge confirmed that the diameters of the dissected arteries had returned to normal (Fig. 4), and antiplatelet medication was discontinued. Changes in the DSA findings over time also confirmed that dissection extended to the posterior cerebral artery. The patient provided consent for publication of this case report.

## Discussion

In this case, bilateral vertebral artery dissection extended to the left posterior cerebral artery, and we identified 2 important clinical issues. First, when intramural hematoma is located in the anterior vessel wall of the basilar artery, brainstem infarction due to perforating artery injury is less likely, and outcomes are better. Second, T1WI is useful for the diagnosis of this rare condition.

Intramural hematoma was located in the anterior vessel wall of the basilar artery in this case. The posterior and lateral surfaces of the basilar artery are rich sources of perforating arteries; however, no perforating branches arise from the anterior surface of the basilar artery [5,6]. Branches and perforating arteries of the vertebral artery also originate from its posterior or lateral surfaces [7]. If the false lumen extends along the anterior vessel wall of the vertebrobasilar artery, branches and perforating arteries are less likely to be involved, which can contribute to better outcomes.

T1WI was useful for the diagnosis of arterial dissection as well as for estimating the distribution of the false lumen in this case. It is often difficult to distinguish infarctions caused by arterial dissection from atherosclerosis, embolism, or other etiologies. Intramural hematoma can be observed as a hyperintense region on T1WI between 7 days and 2 months, which is a useful imaging finding for the diagnosis [8]. Initially in this case, we suspected that infarction in the left posterior cerebral artery territory had been caused by artery-to-artery embolism from the dissected vertebral arteries. Subsequently, we detected intramural hematoma as a hyperintense region on T1WI in the subacute phase. Therefore, it was finally determined that bilateral vertebral artery dissection extended to the left posterior cerebral artery via the basilar artery. To the best of our knowledge, only 1 similar case has been reported to date [9]. TIWI was the deciding factor in the diagnosis of this rare condition.

In conclusion, brainstem infarction is less likely when intramural hematoma is located in the anterior vessel wall of the basilar artery, and T1WI is useful for the diagnosis of this rare condition. T1WI can identify intramural hematoma, which reflects the distribution of the false lumen. It can also be useful in predicting potentially impaired branches and possible symptoms. Further reports should be accumulated to determine the relationship between the distribution of intramural hematoma and symptoms and prognosis.

## Authors' contributions

Masahiro Morishita is responsible for project development, data collection and analysis, literature research and manuscript writing. Koichiro Shindo is responsible for literature research and manuscript editing. Ryunosuke Yoshihara is responsible for data collection. Kohei Ishikawa, Ryota Nomura and Hideki Endo are responsible for manuscript editing. Koji Oka is responsible for data collection and management. Hirohiko Nakamura is responsible for project development and total management.

## Ethical statement

All procedures performed in studies involving human participants were in accordance with the ethical standards of the institution and/or national research committee and with the 1964 Helsinki declaration and its later amendments or comparable ethical standards. The study was approved by the Ethics Committee of Nakamura Memorial South Hospital (No. S2022122901).

## Patient consent

This study was approved by the institutional review board, and informed consent was obtained from the patient.
